# Carbolic Acid as a Remedial Agent

**Published:** 1869-01

**Authors:** W. Kempster

**Affiliations:** Utica, N. Y.


### Miscellaneous.

### Carbolic Acid as a Remedial Agent.

BY W. KEMPSTER, M. D., UTICA, N. Y.

It is not my intention to speak particularly of it as a disinfectant, but rather to offer a few suggestions concerning its use as a therapeutic agent.

Pure carbolic acid is a white crystaline substance, the particles adhering with considerable tenacity, and, after standing for some time, especially if the bottle be frequently opened, becomes slightly deliquescent and more tightly packed together. The two varieties of crystalized acid more generally found in the American market are prepared by Merck, of Darmstadt, and Calvert, of Manchester, England. Merck's preparation has a slight reddish tinge. Calvert's is quite white, having the appearance of snow which has been soaked in water. Merck's contains about 98 per cent. of pure acid, and is slightly more deliquescent than Calvert's, which is pure. Merck's, however, is sufficiently pure for all practical purposes, and is furnished at a lower price.

The first application of this agent, under my own observation, occurred in a case of catarrh, where the discharge was profuse, offensive, and, consequently, very annoying to the patient. Various remedies had been previously tried without success. Hoping to derive advantage from its properties as a disinfectant, it was administered to the patient by inhalation, using one grain to an ounce of water, and conveying the liquid to the affected parts by means of a steam spray-producer. The effect surpassed my most sanguine expectation. It not only relieved the fœtor, but in the course of two or three inhalations changed the character of the discharge and the patient recovered rapidly.

This induced a trial in a second case, not so serious as the first, but still severe, and the result was equally satisfactory, the symptoms all disappearing in the course of four weeks. After the first few inhalations, the patients were instructed in the use of the spray-producing apparatus, furnished with a bottle of the solution (one grain to the ounce) and directed to inhale the vapor for ten minutes at a time, both morning and evening, enjoining upon them not to leave a warm atmosphere for half an hour after each inhalation.

It is used at the present time in the treatment of ozæna, nasal polypi and diseases of the nasal passages in which there is an offensive discharge. Even if it exerted no curative action, its power to correct fœtor would be a great recommendation; but this is not all, it stimulates the ulcerated surface to a healthy action, promotes normal granulation and thus assists in the curative process. This remedy is also employed by some of the physicians who are engaged in the special treatment of throat and lung diseases, particularly French practitioners, who direct that it should be inhaled in combination with other appropriate remedies. They speak highly of its efficacy in case of ulcerated sore throat, chronic bronchitis, and that morbid condition of the mucous surfaces of the air passages which gives rise to a constant expectoration of a muco-purulent material. If a solution of one grain of the acid to an ounce of water does not seem to meet the indication, the quantity may be increased to five grains, or even more, but it is better to begin with a mild solution, gradually increasing the strength until the desired effect is obtained.

My next use of the acid was in a case of scarlatina, where the breath was particularly obnoxious, owing to an ulcerated condition of the throat. A gargle of two grains of the acid to an ounce of water relieved the fœtor at once and apparently proved beneficial. No other gargle or application to the throat was used.

It would seem to be appropriate in cases of diphtheria, a strong solution of the acid being used for a local medicament; its power to correct the foul breath would be an indication for its use, and its astringent and stimulating properties might prove beneficial. In cases of common sore throat (simple tonsillitis) it is found to answer admirably, with the advantage over the ordinary potassa gargles of relieving the "bad taste" and foul breath.

In the State Lunatic Asylum at Utica, it is successfully used to relieve cases of sluggishness of the bowels, accompanied by offensive breath. The dose is a drachm of a solution of one grain to the ounce (which is the house standard.) A striking exemplification of the efficacy of this remedy occurred in the case of a melancholic patient admitted to this asylum. He had for a number of years suffered from attacks of dyspepsia, accompanied with acid eructations and the formations of gas. Latterly these symptoms became continuous. He complained of intense heat and a pain in the stomach; stated that the eructation of fœtid gas had become unbearable; and the same smell emanated from the cutaneous sur face, so that it was offensive to every one in the room. He was at once put into a warm bath, then thoroughly washed with a solution of the acid (gr. v to the ounce.) Internally two drachms of the standard solution were given three times daily for two days. At the end of this time, the breath was sweet, and no unpleasant exhalation from the skin was perceptible. He was also relieved from the painful distension produced by the formation of gas in the stomach and bowels. Whenever he feels the approach of this difficulty, two or three doses of the house preparation relieve him, at once, from this unpleasant and painful complication.

Yeasty stomach, sometimes consequent upon a meal of rich food, which produces flatulence and expulsion of gas, with a tendency to regurgitation, is usually relieved by a drachm or two of the solution above mentioned; this checks the fermentative process. The power it possesses to arrest fermentation would be an indication for its employment in sarcina, but the opportunity has not offered for me to test this. Diarrhœa, produced by eating unripe fruit or other articles which promote fermentation, is speedily relieved by combining a drachm or two of the solution with the usual remedies. As a dentifrice, commingled with myrrh or some aromatic, it removes the odor arising from carious teeth.

As a remedial agent in certain forms of skin diseases it seems to possess decided advantages. A patient applied for something to relieve a disordered condition of the scalp, which had existed for some time. It proved to be a well-marked case of tinea capitis in an advanced stage. The crusts had cracked open, with a straight, smooth fracture, presenting a shining floor, looking as though the scalp had opened and exposed the cranial bones. There were several of these cracks, measuring from a half inch to two inches in length, the principal ones occupying a position over the region of the anterior fontanelle and extending several inches in each direction. Other crusts had formed over the temporal and occipital regions. In order that the acid might be effectually tried, the hair was cut short and the entire scalp washed with a solution of the acid (two grains to the ounce) four times daily. The subsidence of the disease was marked; those crusts in process of formation were checked and the dry, greyish crusts already formed, with those cracked open, were speedily removed. After the wash had been continued for one week, a glycerolate of carbolic acid (strength five grains to the ounce) was applied, which possesses the advantage of being a more permanent preparation. The treatment was commenced January 7th, and at the date of writing (January 28th) the disease has disappeared. No other treatment, either internal or local, was employed. One other case has been mentioned to me, which was even more severe than this, and in which various modes of treatment had been employed without arresting its progress. The treatment mentioned above was resorted to, with an immediate abatement of symptoms and rapid recovery. We have used the glycerolate mentioned in cases of herpes circinatus, with entire satisfaction.

During the month of December, 1867, I was called to see a girl aged four years, who had been taken suddenly ill. The symptoms indicated scarlatina, and, as there were a number of cases in the neighborhood, that diagnosis was made. She was immediately put upon milk-punch and carbolic acid solution, the one-sixteenth of a grain three times daily. I also directed that her face should be washed in water containing a spoonful of the solution (one grain to the ounce) and that the mouth should be sponged out with the same—directing also the use of the commercial acid solution about the house as a disinfectant. At the end of four days the internal administration was discontinued; not because of any unpleasant symptoms, but its continuance did not appear necessary. The mouth-wash, of which the child swallowed a few drops, and all the other applications were continued; the body being anointed with olive oil, tinctured with carbolic acid. From first to last no untoward symptom appeared; the fever subsided on the fifth day. The throat was not very sore; the tongue was relieved of the creamy coat after the third day; there was no offensive breath and the child made a complete recovery. No other treatment was employed. A brother of this child, two years older, who never contracted the disease, and who was with her constantly, had no symptoms of the disorder. His face was washed twice daily in the solution above mentioned.

The medical superintendent of this asylum, Dr. John P. Gray, informs me that in a family of six children, three were simultaneously attacked with scarlatina anginosa. They were put upon a course of treatment similar to the above, the house being thoroughly disinfected, and made a good recovery.

Dr. Gray has spoken to me of a case (sequel to scarlatina anginosa) in which there occurred a very fœtid discharge of ichorous pus from the ears and nostrils of the patient. A mild solution of the acid (two grains to the ounce of water) was thrown into the nares and auditorius externus, with the effect of arresting the sanious discharge and causing its disappearance.

Dr. Bissell states that he has used a solution of carbolic acid—strength two grains to the ounce, the dose being one drachm—as a vermifuge, and has not been disappointed with the remedy. The *oxyuris vermicularis* (pin worm) may be at once destroyed by using as an injection a drachm of the solution to four ounces of water.

Though it was not my intention to speak of this agent as a disinfectant, as it concerns the sick-room directly, yet some remarks may not be inappropriate. Nearly every practitioner has experienced the unpleasant odor emanating from the lying-in room. This may be entirely overcome by the proper use of the solution of commercial acid—a half ounce of which, put into a gallon of boiling water, makes a strong solution—all, indeed, that the water will take up—which, if filtered to remove oily matters, may be thrown about the floor with impunity. Two tablespoonsful at a time are sufficient to disinfect and deodorize a large room, and one-half the quantity is generally sufficient. A few drops sprinkled upon the napkins, and applied to the genitalia externa, will remove the unpleasant, pungent odor which accompanies the lochial discharge, thus exempting the patient from a great source of discomfort. A small quantity of the solution put into the close stool before use, destroys the odor which would otherwise occur. Whenever it has been introduced with these objects in view, it has received the unqualified approval of those most interested.

Carbolic acid at once arrests the development of the lower forms of organic life. It stops the fermentation of yeast, kills microscopic infusoria and cheese mites. Nor does its influence end here. In order to test its destructive power over insect and animal life, I procured a cricket, smeared the inside of a wine-glass with the commercial carbolic acid, and inverted it over the cricket, leaving sufficient space at the bottom to allow a supply of air. Immediately after the glass was inverted, the cricket made violent attempts to escape, lasting two or three minutes. It then staggered about and fell over, had a few severe convulsions, and died. A cockroach was next tried, with the same result; it was from ten to fifteen minutes in the vapor.

A mouse was procured, and put into a wide-mouthed, four-quart bottle. A piece of sponge saturated with two drachms of commercial acid was lowered into the bottle and suspended about two inches from the bottom. Five minutes after the introduction of the sponge, the mouse staggered as if intoxicated, the movements continuing for fifteen minutes, when a short respite occurred. These paroxysms were repeated several times during one hour and a half, then the animal became violently convulsed, the spasmodic action lasting thirty minutes, when it died. Upon examination it was found that the membranes covering the brain and spinal cord were injected, some of the vessels being very large. The lungs were of a slight pink color, many shades above that observed in the normal human lung; they were collapsed. The heart appeared large, and felt hard; upon opening the organ it was found distended with very dark clots, which bulged out as the incision was made.

A full-grown rat was next subjected to the vapor of carbolic acid, and its manifestations were more strongly marked in this than in the former experiments. The animal was a vicious one, exhibiting great ferocity; but in less than one minute after the sponge containing the acid had been introduced, the animal appeared sleepy, and as if intoxicated. Twice the animal reared upon its haunches, as if it desired to climb, but had not the strength to do so, and after each attempt, it fell upon its right side. At the end of forty-five minutes a tremor was observable over the entire body, and it ceased to notice sudden sounds; shortly after this it failed to perceive that it was being handled, and presented all the phenomena of profound anæsthesia. Convulsions followed the tremulousness, which continued to increase in violence until the animal's death, which occurred in one hour and forty-five minutes after the introduction of the sponge. The vessels of the pia mater were found congested, some of them being very much distended. The larger lobes of the brain (cerebrum) presented a greater number of bleeding points than is usually found; the smaller lobes (cerebellum) were highly congested—the vessels being considerably increased in size. The spinal cord appeared exsanguinated in all but the cervical region, which presented a uniform pink blush. The lungs were collapsed and several shades lighter in color than usual. The heart was tense; and, on being opened, a clot bulged out which filled both left auricle and ventricle.

The same experiment has been performed twice since, the result being alike in each case; in the last instance, the convulsions occurred at the end of eighteen minutes; they were more violent in character, and death occurred sooner (fifty minutes.)

A peculiarity was noticed in connection with the convulsive movements of both insects and animals—which was, that the forward legs were first convulsed, the spasm ceasing to a great extent in them, as the posterior members became affected; and also that, as the spasm commenced, the animal fell over upon the right side. —*Canada Medical Journal.*

